# Patient and general practitioner attitudes to taking medication to prevent cardiovascular disease after receiving detailed information on risks and benefits of treatment: a qualitative study

**DOI:** 10.1186/1471-2296-12-59

**Published:** 2011-06-26

**Authors:** Nicola K Gale, Sheila Greenfield, Paramjit Gill, Kerry Gutridge, Tom Marshall

**Affiliations:** 1 School of Health and Population Sciences, University of Birmingham, Edgbaston, Birmingham, B15 2TT, UK; 2 Centre for Ethics in Medicine, Canynge Hall, Whatley Road, Bristol, BS8 2PS, UK

## Abstract

**Background:**

There are now effective drugs to prevent cardiovascular disease and guidelines recommend their use. Patients do not always choose to accept preventive medication at levels of risk reduction recommended in guidelines. The purpose of the study was to identify and explore the attitudes of patients and general practitioners towards preventative medication for cardiovascular disease (CVD) after they have received information about it; to identify implications for practice and prescribing.

**Methods:**

Qualitative interviews with GPs and patients following presentation of in depth information about CVD risks and the absolute effects of medication. Setting: GP practices in Birmingham, United Kingdom.

**Results:**

In both populations: wide variation on attitudes to preventative medication; concerns about unnecessary drug taking & side effects; preferring to consider lifestyle changes first. In patient population: whatever their attitudes to medication were, the vast majority explained that they would ultimately do what their GP recommended; there was some misunderstanding of the distinction between curative and preventative medication. A common theme was the degree of trust in their doctors' judgement and recommendations, which contrasted with scepticism of the role of pharmaceutical companies and academics. Scepticism in guidelines was also common among doctors although many nevertheless recommended treatment for their patients

**Conclusions:**

A guideline approach to prescribing preventative medication could be against the interests and preferences of the patient. GPs must take extra care to explain what preventative medication is and why it is recommended, attempt to discern preferences and make recommendations balancing these potentially conflicting concerns.

## Background

Cardiovascular disease (CVD) is the largest cause of premature death in the UK with a particularly heavy burden on South Asian ethnic groups [1]. There are now a number of drugs that prevent CVD and as a result there are complex questions about whether patients should start medication. Clinical trials have indicated that treatment with statins reduces the probability of developing cardiovascular disease by about 30% of the pre treatment probability [2,3]. A reduction in blood pressure of 10 mmHg reduces probability by a similar amount [4]. This is relative reduction in cardiovascular risk; the absolute benefits of treatment therefore depend on pre treatment probability of cardiovascular disease. A number of studies have indicated that patients prefer to take medication only when the degree of risk reduction is somewhat greater than the risk reduction at which clinicians and clinical guidelines recommend preventive treatment [5-8]. By contrast, in a previous study by the authors of this paper the majority of participants who had accepted an invitation for cardiovascular assessment preferred to take treatment for a degree of risk reduction much smaller than the threshold implied in clinical guidelines. Preferences were influenced by social class, with those from lower socio-economic groups willing to accept treatment for a smaller reduction in risk [9]. However, some remain reticent about medicine taking[10] and it has been observed that whether or not medicines are prescribed for patients is often patterned by social factors, such as age, affluence and ethnicity, although deprivation is often the most overriding factor [11-13]. Information is often, in a commonsensical way, considered to be key to ensuring that prescribing is concordant but, to date, no research has looked at patients' and health professionals' attitudes towards medication for the primary prevention of CVD *after *receiving detailed information on risks and absolute benefit. Our study gave patients and their GPs detailed information about relative and absolute risk reduction in words, numbers and using visual aids on a PowerPoint presentation. The whole presentation took approximately ten minutes to deliver.

The National Institute for Health and Clinical Excellence (NICE) guidelines suggest that, 'Statin therapy is recommended as part of the management strategy for the primary prevention of CVD for adults who have a 20% or greater 10-year risk of developing CVD' [14]. Risk should be calculated using an appropriate risk calculator or by clinical assessment, considering age, co-morbidities and high-risk ethnic groups. It is also recommended that individuals at greater than 20% 10-year cardiovascular risk are treated with antihypertensives if their blood pressure exceeds 140/90 mm Hg and with aspirin if they are aged 50 years or over [15]. Concurrently, the idea of 'concordance and partnership in taking medicines' is high on the health policy agenda [16], based on the principle that clinicians and patients should work together to agree a treatment plan. The NICE guidance states, 'the decision whether to initiate statin therapy should be made after an informed discussion between the responsible clinician and the individual about the risks and benefits of statin treatment, and taking into account additional factors such as co-morbidities and life expectancy'[14]. Concordance and seeking consent for treatment are also considered an ethical duty by the General Medical Council [17]. There is a potential confusion between whether the risk cut-off figure or the individualised decision-making takes priority. To compound this ambiguity for the general practitioner, it is well known that patients often have concerns about side effects, which are muscle pains for statin treatment although these are reported to be infrequent[18] and other concerns about taking medication, such as preference for a more 'natural' approach or that it disrupts their personal or social life[19]. There is some evidence that labelling of individuals as hypertensive or hypercholesterolaemic affects absence from work [20]. This has been the subject of longstanding debate although evidence for the effects of labelling on psychosocial health remains unclear [21,22]. Indeed, concerns have been raised about the ethics of using guidelines for preventative medication as a basis for clinical decision-making and a more critical presentation of the research findings and discussion with patients has been argued for [23]. Research from the social sciences has been instrumental in understanding the complexities of shared decision making and the centrality of the effective two-way communication of information [24-29]. It may be reasonable to assume that providing patients with more (and clearer) information might be useful for promoting a more 'informed' and 'concordant' approach to prescribing.

This study sought to explore the attitudes of both patients and GPs towards medication for primary prevention of CVD *after *they had received detailed information about CVD risk and the absolute benefits of preventative medicine. The qualitative data presented were collected with participants who had been informed of the 'facts' and 'figures' in advance of the interview.

## Methods

The data reported in this paper were collected as part of a larger study on clinician and patient preferences for treatment to prevent heart disease conducted in 2003 [30,31]. The study was approved by South Birmingham [Ref 0782] and North Birmingham REC [Ref 626.01].

Patients identified as likely to be at high coronary risk were invited for cardiovascular risk assessment in their own general practice.

In 13 general practices, patients aged 35 to 74 without cardiovascular disease and not currently taking preventive treatments were identified from electronic primary care records. In order to identify those most likely to be at high risk of cardiovascular disease, patients were ranked in descending order of age (men) and age minus 12 (women). This is because average coronary risk in women is roughly the same as that of men 12 years older [32,33]. This method therefore provided a list of patients ranked by their coronary risk. In each practice, letters were sent to highest risk patients inviting them to attend the practice for coronary risk screening and to participate in the research study. Further letters were sent in descending risk order until sufficient patients attended. This resulted in a sample of participants similar to those likely to take part in a programme of risk screening targeted at those at highest risk.

Prior to assessment they were interviewed face-to-face and asked to consider six scenarios representing different levels of pre-treatment five-year coronary risk. Five-year coronary risk was chosen because in 2003 guidelines used five-year instead of ten-year risk[34]. A five-year risk of 10% is approximately equivalent to a ten-year risk of 20%, which is the current threshold at which treatment is recommended. For each scenario they were asked hypothetically to consider whether they would take preventative medication that reduced their coronary risk by 30% of pre-treatment risk. Because the pre treatment coronary risk varied in each scenario the effectiveness of the drug (absolute benefit) also varied. This allowed us to establish a threshold absolute risk reduction at which participants judged treatment to become worthwhile. Information was presented using decision aids [35]. This showed the number of persons out of 100 who would be expected to suffer a cardiovascular event in the next five years with and without treatment. The information was also presented in the opposite way, stating the number of persons out of 100 who would be expected not to suffer from a cardiovascular event in the next five years with and without treatment. The information was presented both in words and using a vertical bar on a graph where the difference in height of the bar with and without treatment indicating the reduction in risk.

At the end of each presentation, participants were given a 'comprehension test' on the numerical risk information by providing two additional scenarios. For these participants were asked to choose between two otherwise identical treatments that reduced coronary risk by different amounts. Participants who on both occasions preferred the treatment that reduced risk more were judged to have understood the numerical information.

Following assessment by their general practitioner they were interviewed again. At the second interview patients were informed of their real five-year risk and asked again whether if they were offered treatment they would accept it. Information was again presented in words and numbers and visually using decision aids. Preferences were considered to be consistent if their preference at second interview was consistent with the implied threshold risk reduction at first interview. Responses were generally consistent from first to second interview. While age, sex, education and drug treatment history did not affect preferences, lower socio-economic status was associated with preferring treatment at lower risk [36].

In addition to this preference data, supplementary qualitative data were collected from a purposive sub-sample of the patients (reflecting those with demographic characteristics likely to be at high risk) to understand their decisions and preferences in more depth and to get their opinions on the information that was presented to them. At the end of the project GPs in participating practices were also questioned on their preferences in relation to the different hypothetical risk scenarios and asked supplementary qualitative questions. These data were analysed using inductive coding and two analytical frameworks were developed which explored, for example, attitudes to medication and clinical decision making. While separate frameworks were developed for the patients and the GPs, there were some overlapping themes. For the patients, the qualitative data was cross-tabulated in the analytical framework with the quantitative data on whether they understood the information and at what risk level they would accept treatment.

Qualitative data were collected after both the first and follow-up interviews for preference data for a sub-sample of 17 patients. We checked that 17 was a sufficient number for saturation by conducting response validation with 5 additional participants. A single qualitative interview was conducted with all 13 GPs who participated in the study.

The key areas that we wanted to explore with patients were attitudes to taking medication generally and specifically in relation to CVD prevention, whether the risk and benefit information provided in the study was helpful and desirable in decision-making and finally, whose decision the patients felt it should be about whether to take the preventative medication. With the general practitioners, we explored their general views on prevention of CVD, including the role they felt that medication played, and their attitudes to accepting medication themselves if their own risk level was above the threshold at which they would prescribe for their patients.

Transcripts were coded inductively. The key themes that emerged from the data showed a great deal of diversity in the responses for both the patients and clinicians. The data presented here reflect that range of responses and highlight contradictory attitudes within the sample. All comments quoted are anonymised but attributed by employment, ethnic identity, gender and age. For the patients, their actual 5 year risk score (calculated using the Framingham coronary risk equation because at the time of the study this was used to determine treatment eligibility and a coronary risk calculator had been incorporated into electronic primary care databases) and the threshold at which they would accept preventative treatment are included. These last two factors are not included for the GPs as their risk scores were not calculated and they were not asked the question about at what level of risk they would personally accept treatment as part of the quantitative data collection (although it was included in the qualitative topic guide, as above). They were asked at what level they would prescribe for their patients, which is included.

## Results

GP and patient age and ethnic characteristics are described in Table 1.

**Table 1:** Age, gender and ethnic characteristics of the study participants

		Patients	GPs
Age	40-49	1	6

	50-59	2	6

	60-69	8	1

	70-79	6	0

Ethnicity	White British	9	5

	White Irish	4	0

	Asian (Indian or other)	4	7

	Other	0	1

Gender	Male	15	12

	Female	2	1

**TOTAL**		**17**	**13**

### Patients' attitudes to medication for prevention of disease

Overall, patients were cautious about taking medication, many indicating that they preferred to keep medication to a minimum, but that they would if it was necessary for a condition they had or if the doctor recommended it. There was no simple correspondence between the level at which the patients would accept medication and their attitudes to it. Patients who would accept medication at both the lowest and highest levels of risk that they were asked about (3%-40% risk) had a range of positive and negative attitudes.

The largest proportion of interviewees expressed concerns about taking medication, indicating that is was a 'last resort' or that their attitude would depend on whether the condition was 'life-threatening'. Most of these talked about their preference for making lifestyle changes first.

'At the moment I am gradually reducing weight as one of the ways to take the strain off me. I would rather do other things in lifestyle rather than take medication but I've got no objection to medication if that's the only way or the best way'. [Retired designer of engineering tools, White British male, 65, 7% risk, > 30% threshold]

Of this group, some expressed their concern about the effects of tablets on their bodies and expressed concerns about taking medication. The two major concerns were that medication could be addictive and that there might be as-yet-unknown side effects of this medication. These concerns are can be understood in the context of recognised drug side effects, information they are likely to have been exposed to in the media and through their own or their family and friends' experiences, as this respondent's comments show:

'Simple things like taking aspirin; you don't know what you're doing to your stomach... My wife was violently sick when she had our first daughter and that was when they were dishing out Thalidomide... we've got a lovely daughter [but] had [she] have took Thalidomide then what would [have happened?]...Well it might cause something else. One of my neighbours has arthritis and when these arthritic tablets first came out, one of his friends has stomach bleeds taking them. They cause more trouble than they cure!' [Project Manager, White British male, 61, 6% risk, 20% threshold]

Others expressed their concern about medication by expressing their preferences for a more 'natural' approach and talking about the body's self-healing capacity, as these two participants explained:

'[My niece] is into health foods and things... and I agree with her. Tablets are fighting something inside which your body ought to be able to do itself'. [Retired Civil Servant, White British female, 71, 10% risk, 3% threshold].

'If someone offered me an alternative medicine... natural stuff then I would be quite prepared to try those'. [Retired building surveyor, White British male, 71, 12% risk 3% threshold]

A small group of patient respondents expressed unreserved support for medication (although sometimes also explaining that this should happen alongside lifestyle change), for instance:

'Anything to try and prevent, I mean a lot of my friends have died from heart attacks so I'm certainly in favour of trying to prevent it with any means' [Retired manager in motor trade, White British male, 72, 12% risk, 3% threshold].

A number of the patients presented a more ambivalent view, for example:

'I'm not a tablet taker unless there's a real need ... but if I have to take tablets for any reason I will ... just be sensible... lead a sensible life' [Electrical draftsman, White British male, 72, 13% risk, 40% threshold]

'If it is going to extend my life or make it easier for me to do things then great I'm all for it ... [but] whatever you are doing you ... are going to get some slight side effects ... I don't like taking pills or anything' [Retired Toolmaker, White British male, 73, 11% risk, 3% threshold]

### Decision making in preventative treatment

The overwhelming majority, irrespective of their attitudes to medication and even if they had said in the interview that they would only accept treatment at the highest risk levels, explained that, in the final instance, they would do whatever the doctor recommended. In explaining this attitude, they tended to appeal to ideas of the altruism of doctors and their greater knowledge about health and disease.

'The doctor is doing his own job isn't he? We don't know how to cure it. He knows better than us... so you ought to listen to what he says. If I had to go and see him and he says take these tablets then I have got no choice, I should take it' [Retired chemical process worker, Asian Indian male, 64, 15% risk, 3% threshold]

'I think the doctor should make the decision, they know more don't they, that's the way I look at it. They should know best.' [Retired hairdresser, White Irish female, 72, 7% risk, 3% threshold]

One respondent also drew comparisons between his expertise and that of the doctor.

'Because he knows what he is doing. It is for my benefit. If he thinks it's a good idea I would take his advice; it's the same as if he came to me and wanted something in steel or aluminium, I would expect him to follow my advice. I know what I am on about the same as I expect him to know what he is on about.' [Retired toolmaker, White British male, 73, 11% risk, 3% threshold]

It is worth noting that the assumption that prevention of CVD was a desirable outcome was not one shared by all respondents[37].

'I think something has got to go wrong sometime... I think if I had diabetes I would be more worried. I think it [heart attack] is quite a nice way to go-just pop your clogs!' [Project Manager, White British male, 61, 6% risk, 20% threshold]

### The information provided of risks and effectiveness of treatment

The attitudes to the information presented did not necessarily correspond to whether the respondents understood the data, however, there were some trends noted.

One respondent [Retired toolmaker, White British male, 73, 11% risk, 3% threshold], who did not pass the comprehension questions, said that he would 'prefer to know why' and 'as long as you've got the information to look at, he can recommend it to you. After all, he is the expert, you're not. At least you've got some idea of what's likely to happen... To a lot of people my age, percentages don't mean a thing to them but the way you have presented it, it is so easy to find'. However, he pointed out 'looking at it from the doctor's point of view it would be time consuming and they are under tremendous pressure'. The interviewer asked him 'what about from your point of view' and he responded, 'You could do with something far simpler, if it was just the doctor talking to me.'

One respondent [Retired factory processor, White Irish male, 68, 11% risk, 3% threshold], who had also failed the comprehension question stated that he found the information irrelevant: 'I just go along with the doctor at all times. I'm not impressed with graphs and figures.' However, he also demonstrated an understanding that the information that was underpinning medical practice was not straightforward: 'Occasionally they are wrong because doctors differ and patients die'.

Others (who all passed the comprehension questions) liked the idea of joint decision making:

'I do understand the statistics that are shown... and it is like making a joint decision, otherwise [you'd] just leave it to the doctor. It's like a team... I like the statistics.' [Clothing wholesaler running own business, Asian Indian male, 48, 2% risk, > 30% threshold]

'It makes you feel that you're doing something for yourself and you're helping yourself and it's more or less your decision as well. You're not in someone else's hands.' [Retired labourer in building trade, White Irish male, 69, 6% risk, 3% threshold]

'Most professional people are experts in their own field so you have to be led by them but you don't have to be led blindly, do you?' [Retired sales manager, White British male, 65, 8% risk, 3% threshold]

Some were very critical of the use of the findings on the level of benefit of the preventative treatment to justify putting people on medication. These responses echo the concerns of some clinicians in our sample (see below). The first three of these critical voices came from patients who passed the comprehension questions; the final one had not passed the questions.

'I would need to have a very obvious benefit [of taking preventative medication] to persuade me to do anything about it now.' [Retired electrical draftsman, White British male, 72, 13% risk, > 30% threshold]

'With a low improvement rate I felt that isn't really worth the inconvenience of taking the tablets' [Retired designer of engineering tools, White British male, 65, 7% risk, > 30% threshold.

'I think it is silly because 6% is hardly anything. If... the doctor said well I think you'd better have surgery because chances of coming out are 6%... It's a very small percentage you know.' [Clothing wholesaler running own business, Asian Indian male, 48, 2% risk, > 30% threshold]

'Simple information such as a difference of 2% well it's not worth having the patient worry about it. I suggest the doctor doesn't tell the patient until it gets to a percentage where the difference counts and I think that is about 15, 20, 30 going up that way. I think the figures will worry the patient more unnecessarily' [Security guard, White British male, 60. 11% risk, 15% threshold]

For some, this wariness was reinforced by a lack of faith in research findings, due to a mistrust of academia and/or the pharmaceutical industry. The first of these quotes comes from a respondent who was not asked the comprehension questions; the second had not answered them correctly. Both had previously stated that they would accept treatment at the lowest level of risk.

'Well I'm giving money or someone is giving money to the big pharmaceutical companies and that's all they are interested in-making money. Whether the things are suitably made, properly made and all that, I'm not sure about.' [Retired Civil Servant, White British female, 71, 10% risk, 3% threshold]

'Well if you've been given information, it can be false I think. I think people are chancing their arms sometimes you know, with all these Professors and research, I think that on occasion they are wrong and it makes you a bit dubious you know so in my case I am going with the tried and trusted.' [Retired factory processor, White Irish male, 68, 11% risk, 3% threshold]

### GP attitudes to preventative medication

Most of the GP respondents (n = 10) had a balanced attitude to prevention of CVD, usually stating that lifestyle changes were the preferred place to start to support prevention or indicating that the two things needed to happen alongside each other. Many of these drew on the language of concordance to support their views. As one GP explained,

'I try to have a discussion with people to find out how much they want to use lifestyle modification and I think in situations it is very important to have the patient try the lifestyle to see if it will work and then treat them, to give them the option... I try to determine their preferences'. [GP, White British male, 44, prescribe at 20% threshold].

Two of the GPs expressed grave concerns about the value of preventative medication, citing side effects and the wastage as the key reasons for this.

'My view is that statins are greatly over rated, that the numbers needed to treat (NNT)... of the best trials is effectively equivalent to saying that 95% of treatment with statins is wasted-95%-this is a scandal which is fuelled by the interests of the pharmaceutical industry and the vested interests of the medical profession... [this is] an enormous cost to the taxpayer...Now one of the problems... is the new contract which is extremely unethical because... people who prescribe these medications will earn more which is very sickening.' [GP, Asian Indian male, 43, prescribe at > 30% threshold].

Only one doctor [GP, Asian Indian male, 62, prescribe at 3% threshold] said that he would always recommend preventative medication to their patients, saying 'I don't take the slightest risk with someone else's life' but also (as discussed further below) said that he would only ever take curative medication himself.

### GP attitudes to accepting preventative medication themselves

Asking GPs if they would accept preventative medication themselves, if their risk score was above the cut-off that they had indicated that they would use for their patients, produced mixed results. A few stated that they would accept the same levels of risk as their patients, but the majority were keen to 'try very hard the lifestyle modification before getting into any drugs' [GP, White British male, 44, prescribe at 20% threshold]. One who had indicated that they were anti-medication, stated that they 'practice what I preach' and so went to the gym regularly to stay fit, they added, 'Its really a small chance of these poisons helping so I won't take them' [GP, Asian Indian male, 43, prescribe at > 30% threshold], while another stated 'If it is for prevention, I would not take any medication' [GP, Asian Indian male, 59, prescribe at 3% threshold]. Some talked about their individual risk scores and emphasized that all decisions would depend on individual cases, such as ethnicity and family history. A number dodged or refused to answer the question. For instance:

'I think that is a different question. I would say that is a general point, personally I have not had my cholesterol tested. My personal views are that I don't think it is dreadful if the doctor smokes. I don't and never have, but I don't think you need the thing of the doctor not smoking, exercising etc in order to give advice to people' (GP, White British male, 56, prescribe at 15% threshold].

For the doctor mentioned in the previous subsection, who was supportive of preventative medication for his patients, said 'Myself it is different, we treat patients more medically than we treat ourselves... Pain and disability would need to be there before I would take drugs.' [GP, Asian Indian male, 62, prescribe at 3% threshold].

### GP attitudes to guidelines and targets/erosion of professional autonomy

Although we did not specifically ask for their views on policy or targets, in a number of interviews, the issue emerged spontaneously. In general, those who mentioned this were concerned that CVD and other chronic diseases were 'an epidemic' and felt that the targets for reducing heart disease were either 'unrealistic' or that the policy was inappropriately focused on medication rather than lifestyle change. One GP explained:

'I feel that prevention is the ideal and whether you treat something with acupuncture, homeopathy or whatever the whole idea really is that the person need to get their act together... smoking, obesity, lack of exercise, I think this is far more important than the goal at the moment, the protocol, which is very much prescribing medication' [GP, White British male, 52, prescribe at > 30% threshold].

'I think patients need to take responsibility for their lifestyle rather than rely on the doctors to treat the problems' (GP, Middle Eastern male, 55, prescribe at 30% threshold].

Another GP shared his strong beliefs on the ethics of the Quality Outcomes Framework (QOF) and the General Medical Services (GMS) contract:

'One of the problems is the new contract which is extremely unethical because there is an incentive for doctors to prescribe these... people who prescribe these medications will earn more which is very sickening... My philosophy... is, very simply, consensual prescribing.' [GP, Asian Indian male, 43, prescribe at > 30% threshold].

## Discussion

The unique contribution of this paper is related to the discussion of attitudes to decision making *after *receiving detailed and balanced information about the risks and benefits of preventative treatment for CVD. Two principal findings emerge: first, about whose decision it is to commence treatment and, second, what role the information plays in that decision.

### Deciding to medicate or not: whose decision is it?

The issue of preventative medication, both amongst the patient and clinician population, is clearly controversial [38-44] and our findings reflected this through the diversity of responses; indeed, many responses directly contradicted others. Both groups expressed some concerns about medication and generally there was a preference for lifestyle changes, although a significant minority of both patients and GPs expressed extreme views either for or against prescribing medication. Where patients expressed clear favourable or unfavourable views, in principle, to preventative medication, these did not show a clear correspondence to the risk thresholds at which they said they would accept medication. To compound this, the overwhelming majority of patients said that, in the final instance, they would do whatever the doctor recommended. Interestingly, however, the trust that patients showed in their GP's opinion was not extended beyond that personal relationship.

Patients' confidence in their GPs' judgements contrasted with their distrust of distant or unfamiliar information sources. Both GPs and patients indicated concern about other interested parties (academics, pharmaceutical industry and guideline authors) trying to influence their decision-making. There is a literature on 'disease mongering' [45] which may help explain this attitude. For the GPs, these trends may also be related to threats to their professional autonomy. Many patients expressed significant concerns or scepticism about the value of preventative medication. These concerns were also well represented amongst the clinicians, reflecting wider debates in the medical profession on statins and other drug treatments, such as aspirin [46].

For some GPs, there is a striking discordance between prescribing for their patients and preferring lifestyle changes for themselves. The assumptions and values that underlie GPs' decisions to treat patients more medically than they would themselves bear further investigation in future studies. However, it is important to point out that there may be few satisfactory alternatives. There is little evidence to suggest that lifestyle advice has any significant effect [47] although it is worth noting that it is difficult to design a rigorous trial to evaluate this kind of intervention.

This study was limited by its location in one urban area of the UK (both ethnically and economically diverse) and both patients and GPs from other geographical regions may have different experiences. In addition, the vast majority of our sample were men (both the GPs and patients), and while this reflects the demographics of the local GP population and the population at risk of CVD, it would be useful in future to explore women's perspectives more thoroughly. It would be of interest to explore quantitatively the relationship between GP attitudes to preventative medication and prescribing rates in their practice.

### The role of information in the decision making process

Despite providing participants with detailed descriptions and visual representations of risks and benefits of treatment, the data collected did not provide evidence to support a direct relationship between the information and the outcome of decision-making. This adds a new dimension to the existing literature. It has long been argued that clinicians' decisions are influenced by the way in which numerical information is framed. Clinicians are more likely to offer treatment when benefits expressed as relative risk reductions than absolute risk reductions [48-50]. Similar observations about relative risks have been made in relation to patients' preferences [51]. Patients are also more persuaded to take treatment when effects of treatment are framed in terms of reduced potential losses (fewer adverse events or deaths) rather than potential gains (higher chance of healthy survival). Better understanding of information is associated with a greater caution in relation to taking treatments. The information we provided (described above) balanced these potential biases. While patients largely welcomed the information, because they felt more involved, they were quite clear that it did not affect whether they would accept treatment. They would accept the doctor's recommendation. It could be reasonably hypothesized that this feeling of involvement would increase adherence over time to treatment, but this was beyond the scope of our study. It would be interesting to conduct a longitudinal study to track whether the modes of communication of risk information and the prescribing decision (including commitments to any lifestyle changes) were maintained over time. In addition, this kind of study might be very useful to repeat as new approaches are developed to provide information to patients on any condition and its treatments.

## Conclusions

The tendency for patients to comply with doctors' decisions, even when they are not in line with their own preferences [52] is a significant concern in decision making around starting preventative medication. Doctor-patient communication about drugs is not always effective [53] and it is common to find misunderstanding between patients and their GPs about preferences for and experiences of medicine taking [54]. This study underlines the responsibility of general practitioners to ensure that they are acting in the patient's best interests, although there remains an outstanding potential conflict between adhering to national guidelines and prioritising patient's preferences. Our findings showed that however clearly the information was presented, there was still some confusion between preventive and curative medication. This may be the result of a culturally-informed assumption that medicine primarily has a curative role. It is important that the patient understands what preventative medicine is and that it is possible that it may make no difference to whether or not they suffer from heart disease. Providing basic information about risks and benefits can help with this, yet the GP's recommendations are the most influential factor in patient decision making. However, although trusted by their patients, GPs see themselves as subject to pressure from other sources. Although it is beyond the scope of our study, it is also worth noting that although patients may agree to being put on medication at the consultation, they may then not adhere to treatment long term [10,55].

Incentive payments such as those introduced to UK general practice in the Quality and Outcomes Framework of 2004 are an added complication. Payments to practices are linked to achievement of blood pressure targets in hypertensive and diabetic patients and coronary heart disease patients receiving specific preventive drugs such as aspirin, statins, angiotensin converting enzyme inhibitors and beta-blockers. There is an implicit tension between patient choice and achieving targets and GPs seem to be more willing to follow recommendations when deciding for their patients than when deciding for themselves. Calls for doctors to engage in partnership decision-making around preventative medication may be idealistic at this time [23]. Our findings have starkly indicated that, as in the screening debate, the interests of the whole population, and the interests and preferences of particular individuals may not be the same. At a clinical level, however, decision making must clearly be on a case-by-case basis, taking into account the social and cultural context in which the patient may have to integrate tablet taking, their medical history and their preferences.

## Abbreviations

CVD: cardiovascular disease; GP: general practitioner; NICE: National Institute for Health and Clinical Excellence.

## Competing interests

The authors declare that they have no competing interests.

## Authors' contributions

NG led the analysis of the data, wrote the first draft of the paper and coordinated contributions to writing the article; SG designed the data collection instruments and assisted with the data analysis; PG was involved in the design of the study; KG collected the data and was involved in the analysis; TM led the study and was involved in the analysis. All authors commented on the article and approved the final version.

## Pre-publication history

The pre-publication history for this paper can be accessed here:

http://www.biomedcentral.com/1471-2296/12/59/prepub
